# Rapid digitization to reclaim thematic maps of white-tailed deer density from 1982 and 2003 in the conterminous US

**DOI:** 10.7717/peerj.8262

**Published:** 2020-01-22

**Authors:** Brice Hanberry, Phillip Hanberry

**Affiliations:** 1 Rocky Mountain Research Station, USDA Forest Service, Rapid City, SD, United States of America; 2 Missouri Resource Assessment Partnership (MoRAP), University of Missouri, Columbia, MO, United States of America

**Keywords:** eCognition, GIMP, Herbivory, Historical geography, Object-based image analysis

## Abstract

**Background:**

Despite availability of valuable ecological data in published thematic maps, manual methods to transfer published maps to a more accessible digital format are time-intensive. Application of object-based image analysis makes digitization faster.

**Methods:**

Using object-based image analysis followed by random forests classification, we rapidly digitized choropleth maps of white-tailed deer (*Odocoileus virginianus*) densities in the conterminous US during 1982 and 2001 to 2005 (hereafter, 2003), allowing access to deer density information stored in images.

**Results:**

The digitization process took about one day each per deer density map, of which about two hours was computer processing time, which will differ due to factors such as resolution and number of objects. Deer were present in 4.75 million km^2^ (60% of the area) and 5.56 million km^2^ (70%) during 1982 and 2003, respectively. Population and density in areas with deer presence were 17.15 million and 3.6 deer/km^2^ during 1982 and 29.93 million and 5.4 deer/km^2^ during 2003. Greatest densities were 7.2 deer/km^2^ in Georgia during 1982 and 14.6 deer/km^2^ in Wisconsin during 2003. Six states had deer densities ≥9.8 deer/km^2^ during 2003. Colorado, Idaho, and Oregon had greatest increases in population and area of deer presence, and deer expansion is likely to continue into western states. Error in these estimates may be similar to error resulting from differential reporting by state agencies. Deer densities likely are within historical levels in most of the US.

**Discussion:**

This method rapidly reclaimed informational value of deer density maps, enabling greater analysis, and similarly may be applied to digitize a variety of published maps to geographic information system layers, which permit greater analysis.

## Introduction

Researchers increasingly are developing and improving tools that can be applied to a range of topics. Object-based image analysis is a relatively new avenue of research, with few publications referencing use before 2010 (Chen et al., 2018). Current application and development of object-based image analysis primarily is for remote sensing objectives of land use and cover mapping, but this analysis can be applied to achieve other objectives. Indeed, Trimble, a geospatial company, acquired eCognition object-based image analysis software from a medical imaging company (Trimble, 2010).

The GIS community has not yet taken full advantage of this powerful tool, perhaps due to unfamiliarity (Chen et al., 2018) and many potential applications are unrealized, including research of historical maps and geographies using object-based image analysis for rapid digitization. This digitization method may not be novel, but it is seldom disseminated as an alternative to rigorous and monotonous digitization by hand for archived materials. Early adopters include Sharma (2006) and Bracke, Miller & Kim (2008), who published similar techniques for digitization of old maps. Recently, Liu et al. (2018) used object-based analysis to digitize a civil war map and aerial photos.

Currently, there are many thematic maps that are not digitized and yet may provide a valuable resource for research. Thematic maps display spatial variation of a specific variable, for example, demographic data, in a geographic area. Choropleth maps are thematic maps with colored or shaded measurements of the thematic variable aggregated over defined spatial units, such as counties. For example, the Southeastern Cooperative Wildlife Disease Study (https://vet.uga.edu/scwds/range-maps) assembled a map of estimated white-tailed deer (*Odocoileus virginianus*) density during approximately 1982 for the conterminous United States, based on information from state wildlife agencies that generally is estimated from harvest data and deer surveys (Webb, 2014). Equally, the Quality Deer Management Association (QDMA; Adams, Hamilton & Ross, 2009) generated US maps of deer density during 1994 to 1999 and 2001 to 2005, although some states did not provide information during 2001 to 2005. According to Adams, Hamilton & Ross (2009), the maps have been used regularly as a reference to compare relative deer densities among states. Rather than automating the procedure with the assistance of software programs, the most common approach used to digitize maps is a labor-intensive method of drawing objects or polygons, editing, and selecting, which limits the digitization of non-digital images. For example, Walters, Woodall & Russell (2016) used this approach to digitize the 2001 to 2005 deer density map, but limited their efforts to the eastern region of the United States and did not publish their methods.

New methods and software applications make it possible to rapidly convert map images to digital format. However, automation is not widely applied to accelerate digitization of map images and remains an unused alternative to labor-intensive digitization of objects. Our objective was to demonstrate the application of eCognition software to automate digitization of published images, primarily through identification and aggregation of ‘image objects’ that shared the same colored or shaded measurements of the thematic variables. Time inputs still are required for georeferencing, sample selection, rule set creation, and manual error correction. We also present the reclaimed ecological information stored in images, providing an analysis of change in deer density over time and discussion of the potential effects of changing deer densities over time.

## Methods

**Figure 1:**
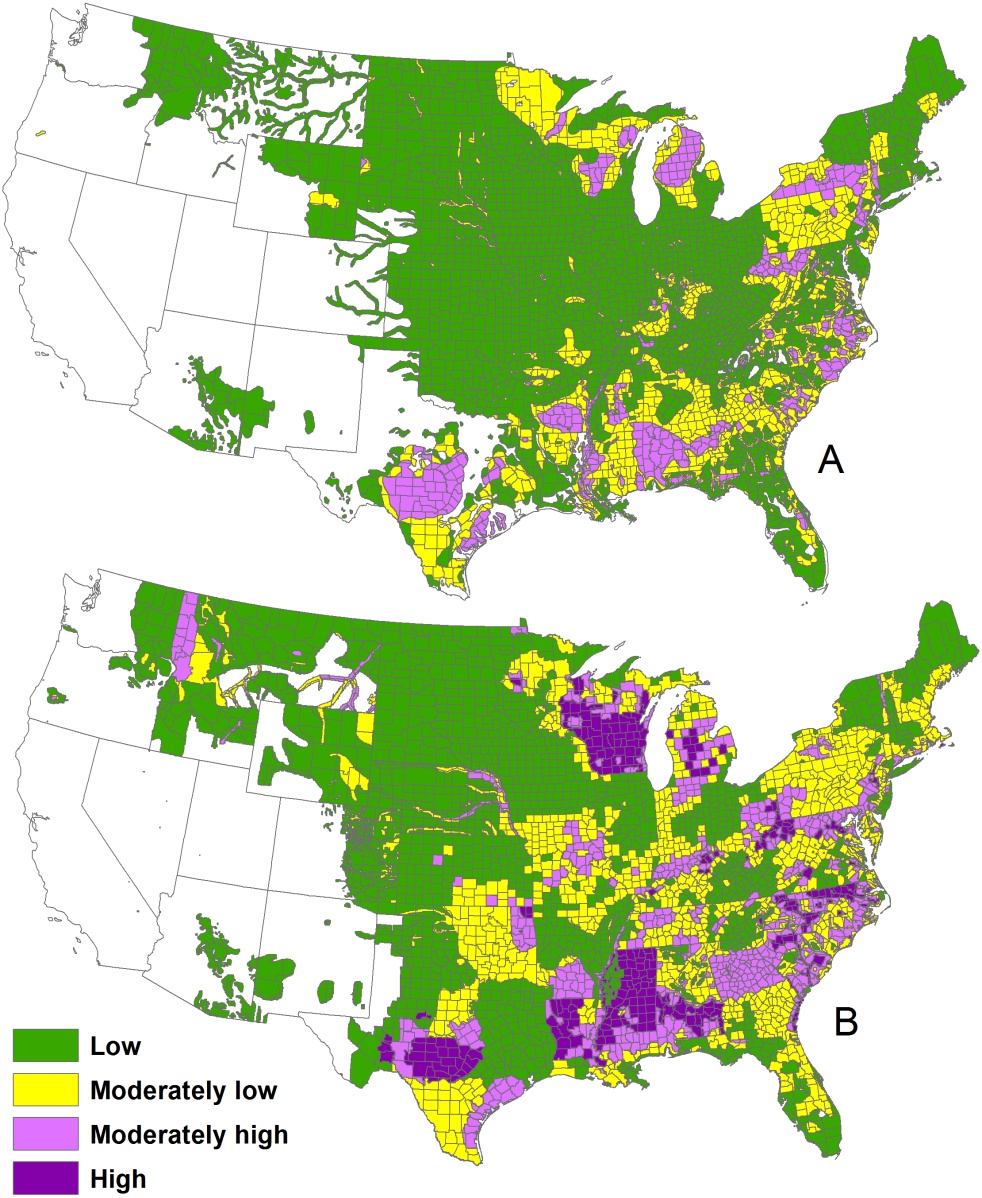
The 1982 deer densities (A) and 2001–2005 deer densities (B) in the conterminous United States.

We digitized the Southeastern Cooperative Wildlife Disease Study 1982 map of deer density (https://vet.uga.edu/scwds/range-maps) and the Quality Deer Management Association 1994–1999 (hereafter, 1996), and 2001–2005 (i.e., 2003) maps of deer density (Adams, Hamilton & Ross, 2009) for the conterminous United States. The maps grouped deer densities into four colored classes: <5.8 deer/km^2^, 5.8–11.6 deer/km^2^, 11.6–17.4 deer/km^2^, and >17.4 deer/km^2^ (Fig. 1). Because the 1996 and 2003 maps were very close in time, we used the 1996 map to fill information in the 2003 map that was not provided by state wildlife agencies, primarily in Nebraska and Colorado (Adams, Hamilton & Ross, 2009).

The images were JPG and PNG files, which contained a stack of red, green, and blue bands. We imported these images directly into ArcGIS Pro (v2.2, ESRI, Redlands, CA). We set the projections to Albers equal area conic USGS version. We then georeferenced the images to a United States county layer using a third order polynomial transformation.

Once georeferenced, we imported the layers into eCognition (v9.3.2, Trimble, Westminster, CO) and built image objects (i.e., object-based image analysis delineates homogeneous pixels into shapes or polygons), using multi-resolution segmentation with all bands weighted equally. Because the images had poor resolution, the borders mixed in color, and thus, we applied a small scale factor to minimize mixed color objects and to delineate borders of deer density classes.

To determine the deer density classes of the image objects, we applied random forests classification, which involves combination of many classification trees and random samples to produce a strong model. We manually assigned deer density classes and no data values to a sample of image objects, which was the training input for a random forests classification (R Core Team, 2018; Supplement 1 provides example R code) along with the mean and standard deviation of each band color (red, green, and blue bands) to inform the model. Random forests used the relationship between the known deer densities and color variables to automatically assign deer density classes to all image objects. Random forests classification of image objects appeared to be both faster and produce a better result that required less manual correction than nearest neighbor classification in eCognition, based on classification of deer densities and our experience for remote sensing work, although we did not formally document processing and correction times.

To correct issues specific to these images, we used rule-based classifiers. County names and borders were colored black and we reclassified black objects to a colored deer density class using a process in eCognition called ‘assign class algorithms’. Black objects ≤ 5 pixels were reclassified to the surrounding majority colored deer density class. We manually corrected any errors that persisted at the border of two colored deer density classes.

An alternative free option to eCognition software that provides a more serviceable product than multi-purpose GIS software is GNU Image Manipulation Program (GIMP v2.1.0.2; the GIMP team). For this software, after georeferencing the image, we selected each color and continued to select until we reached the full color range. We exported each color into the georeferenced layer, resulting in multiple copies. In ArcMap (v10.6, ESRI, Redlands, CA), we extracted by attribute for the raster value of 255, which was the information selected in GIMP. We filled in holes within colors using the union function and then erased any holes that were of a different color.

We used an approximate conterminous US population estimate of 30 million at year 2000 (Webb, 2014) to calibrate population estimates. The lowest value for each density class (i.e., 5.8, 11.6, 17.4 deer/km^2^) and a value of 1.85 deer/ km^2^ for the low density class was necessary to generate this population value from the 2003 map. We then estimated population and density for the 1982 map. The best available estimates for comparison were 15 million deer by 1978 and 26 million deer by 1993 (Miller, Muller & Demarais, 2003) for this map.

In order to compare spatial change at the county level, we determined the percent area of each deer density class by county. We assigned the majority, or greatest percent area, to each county. In order to prevent errors in counties with two or more deer density classes, we retained only counties with a clear majority of >25 percentage points for the majority deer density class compared to the next most abundant deer density class (for example, the majority class of 45% of the area compared to a class of 20%). We also excluded counties with partial information of less than 25% of the county area reported to be in any deer density class.

To provide an estimate of error, we located archived 2005 deer population estimates from the Quality Deer Management Association (Adams & Ross, 2015) for 25 US states and 2001 to 2005 deer population estimates from the Southeast Deer Study Group proceedings (Southeast Deer Study Group, 2002–2006), which is limited to 16 southeastern states. We compared reported estimates to our values derived from the 2003 map. State agencies provided all estimates.

## Results

After testing the procedure, the digitization process took about one day each for these images to complete the data processing steps of georeferencing, building image objects, random forest classification, and correcting errors, of which about two hours was computer processing time. Computer processing time will differ due to factors such as resolution and number of objects, that is, heterogeneity of the image. Errors that required manual correction resulted from borders, text labels, and poor resolution between colors. More colors and indistinct colors will increase manual correction time, and it may not be possible to have an acceptable product relying on automation for low contrast or complex symbology. In GIMP, processing time was minor compared to eCognition but more manual correction was required after automation to fill in areas without color.

Deer were present in 4,752,100 km^2^ (about 60% of the conterminous US) and 5,561,500 km^2^ (70% of the area) during 1982 and 2003, respectively. Population and density were 17,148,500 and 2.2 deer/km^2^ during 1982 and (as calibrated) 29,928,700 and 3.8 deer/km^2^ during 2003. The value of 17 million deer for the 1982 map is in line with 15 million deer by 1978 and 26 million deer by 1993 described in Miller, Muller & Demarais (2003) and based on best available population estimates. In areas where deer were present, deer densities were 3.6 deer/km^2^ during 1982 and 5.4 deer/km^2^ during 2003.

**Figure 2:**
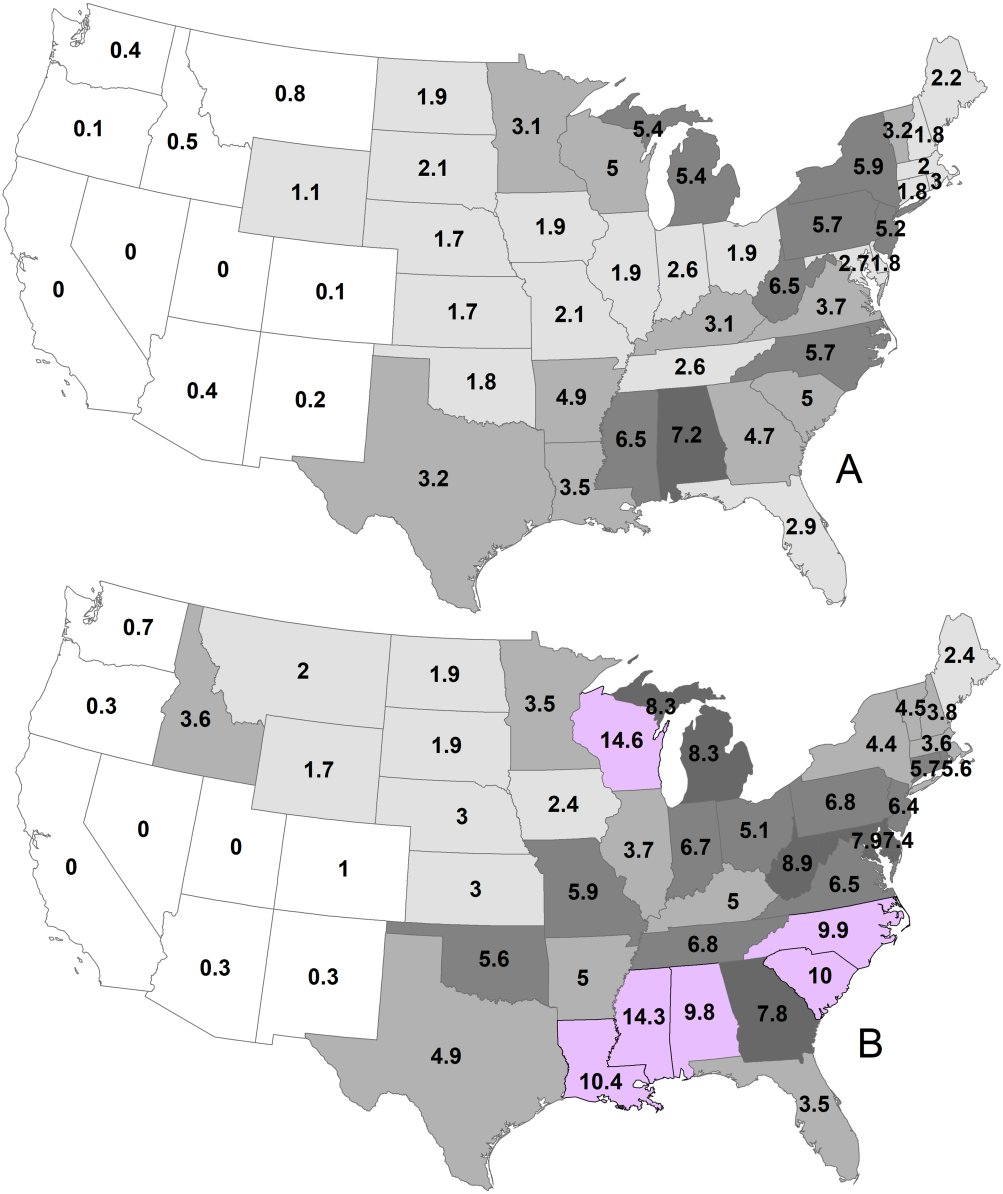
Mean statewide deer densities (deer/km^2^) during 1982 (A) and 2003 (B).

**Figure 3:**
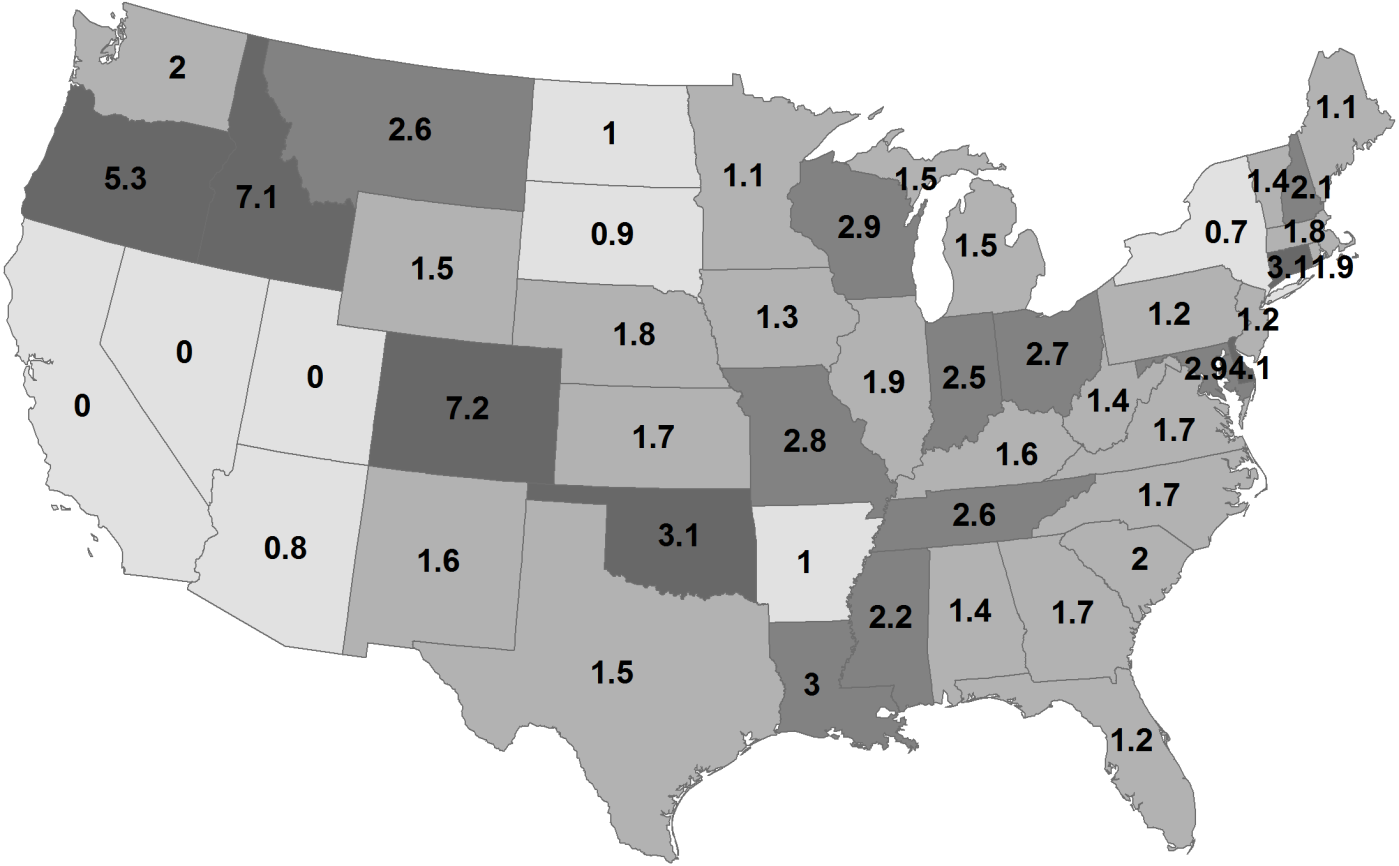
Change in deer population (2003 population/1982 population) by state.

By state, the greatest density was 7.2 deer/km^2^ in Georgia during 1982 (Fig. 2; Table 1). Conversely, 11 states had densities greater than this value during 2003, ranging up to 14.6 deer/km^2^ in Wisconsin. The greatest population increase (i.e., 2003 population/1982 population >7) occurred in Colorado and Idaho (Fig. 3). A few states had decreased deer populations, which may result from reporting differences rather than population changes due to ecological factors. Greatest area expansion (i.e., 2003 area/1982 area ≥ 5) occurred in Colorado and Oregon, although area of more eastern states had complete or nearly complete deer presence by 1982.

**Table 1:** Population, statewide density (deer/km^2^), and area (km^2^) where present of deer during 1982 and 2003 by state.

State	1982			2003		
	Population	State density	Area	Population	State density	Area
Alabama	960,480	7.2	133,410	1,313,020	9.8	133,720
Arizona	107,170	0.4	57,930	87,110	0.3	47,090
Arkansas	680,320	4.9	137,670	686,810	5.0	137,720
California	0	0.0	0	180	0.0	100
Colorado	37,750	0.1	20,410	272,440	1.0	118,400
Connecticut	23,920	1.8	12,840	73,340	5.7	12,900
Delaware	9,470	1.8	5,110	38,880	7.4	5,250
Florida	444,180	2.9	130,900	525,210	3.5	147,510
Georgia	720,390	4.7	151,590	1,191,410	7.8	152,390
Idaho	111,610	0.5	60,330	789,900	3.6	171,590
Illinois	275,790	1.9	145,780	537,490	3.7	145,910
Indiana	246,460	2.6	93,670	627,170	6.7	93,700
Iowa	270,110	1.9	145,740	353,060	2.4	145,740
Kansas	367,210	1.7	198,490	637,370	3.0	213,100
Kentucky	320,540	3.1	104,660	518,650	5.0	104,660
Louisiana	424,450	3.5	94,390	1,273,790	10.4	121,160
Maine	185,920	2.2	81,880	209,420	2.4	83,940
Maryland	72,430	2.7	25,510	211,590	7.9	22,830
Massachusetts	42,670	2.0	19,610	77,390	3.6	20,440
Michigan	812,250	5.4	146,750	1,250,570	8.3	149,700
Minnesota	683,410	3.1	214,220	759,990	3.5	218,500
Mississippi	803,860	6.5	123,270	1,767,950	14.3	123,470
Missouri	378,350	2.1	180,550	1,071,110	5.9	180,550
Montana	289,870	0.8	156,620	763,120	2.0	281,270
Nebraska	335,290	1.7	165,220	597,830	3.0	200,340
Nevada	0	0.0	0	270	0.0	150
New Hampshire	44,350	1.8	23,950	92,070	3.8	23,910
New Jersey	104,950	5.2	17,840	129,420	6.4	19,880
New Mexico	61,760	0.2	33,390	96,890	0.3	52,370
New York	749,340	5.9	123,810	554,740	4.4	12,6570
North Carolina	737,340	5.7	119,680	1,285,590	9.9	127,870
North Dakota	350,370	1.9	182,230	338,820	1.9	183,100
Ohio	200,410	1.9	106,610	547460	5.1	106,960
Oklahoma	328,280	1.8	175,180	1,014,740	5.6	181,030
Oregon	14,570	0.1	6,660	76,840	0.3	33,030
Pennsylvania	663,490	5.7	116,420	798,120	6.8	117,320
Rhode Island	8,590	3.0	2,090	15,990	5.6	2,550
South Carolina	406,640	5.0	77,820	807,740	10.0	80,400
South Dakota	410,140	2.1	199,720	369,770	1.9	199,730
Tennessee	285,840	2.6	109,140	736,820	6.8	109,160
Texas	2,219,730	3.2	345,460	3,385,920	4.9	586,980
Utah	0	0.0	0	30	0.0	20
Vermont	78,570	3.2	24,090	111,630	4.5	24,820
Virginia	390,180	3.7	102,110	682,330	6.5	103,320
Washington	62,510	0.4	33,790	126,860	0.7	67,970
West Virginia	405,070	6.5	62,760	561,530	8.9	62,760
Wisconsin	730,900	5.0	143,970	2,119,670	14.6	145,180
Wyoming	289,800	1.1	139,050	440,690	1.7	174,630

**Figure 4:**
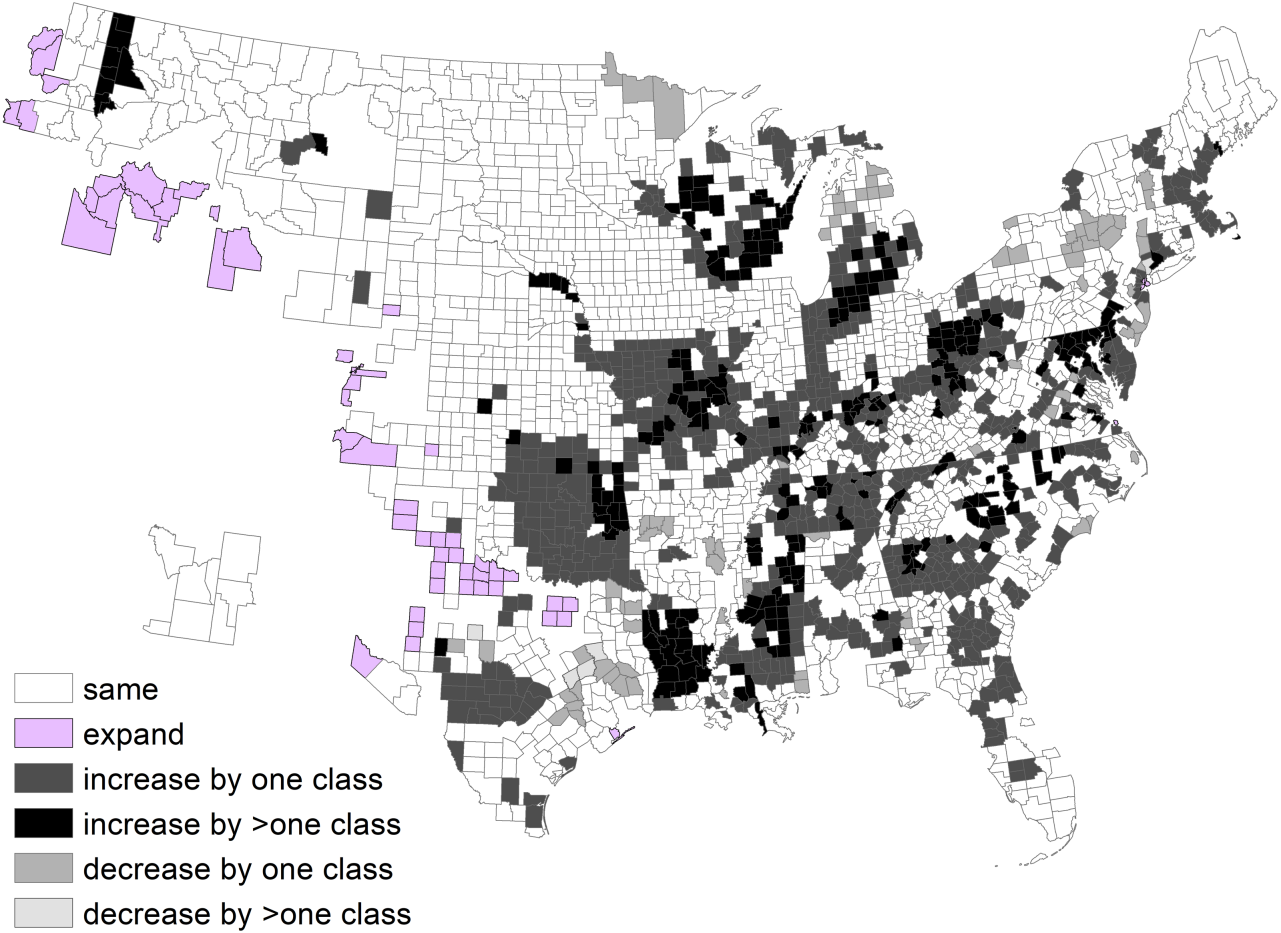
Change in deer density classes by county between 1982 and 2003, after exclusion of counties without a clear majority classes.

We determined changes at a county scale. The low density class was 69% of area during 1982 and 52% of area during 2013. About half of the counties remained in the same density class, 30% of counties increased by one class, and 12% of counties increased by >1 density class (Fig. 4).

Comparison between the reported estimates for 25 US states from the Quality Deer Management Association (Adams & Ross, 2015) and the 2003 map of deer densities was very close for all 25 states combined, with very similar population totals of 20,354,137 and 20,429,770, a difference of about 75,600 (Table 2). However, mean absolute error (or difference) was about 235,000. Most reported deer density estimates in 16 southeastern states (Southeast Deer Study Group, 2002–2006) were greater than the 2003 map of deer densities and overall for the Southeast, the reported estimates were greater by a factor of 1.09 (18,500,000 vs. 17,000,000 deer). The mean absolute error (or difference) between mean deer densities reported by the Southeast Deer Study Group and estimated from the 2003 map of deer densities was about 250,000 (Table 3). Although this value is high, reported mean deer densities also ranged considerably from year to year, for example, changing by 250,000 individuals. For some states, reported deer densities did not change at all. In addition, although state agencies provided all estimates, reported estimates from the Quality Deer Management Association (Adams & Ross, 2015) varied in some states from the Southeast Deer Study Group, resulting in a mean absolute error of 175,000 for the 13 states with overlapping information. Thus, error in the 2003 map of deer densities may be little worse than error due to differential reporting by state agencies.

**Table 2:** Comparison between estimates reported by Quality Deer Management Association (Adams & Ross, 2015) and the 2003 map estimate.

State	2005 QDMA	2003 estimate	Difference	Quotient
Alabama	1750000	1313020	436980	1.33
Delaware	45000	38880	6120	1.16
Georgia	1470000	1191410	278590	1.23
Kansas	400000	637370	−237370	0.63
Kentucky	847911	518650	329261	1.63
Louisiana	750000	1273790	−523790	0.59
Maine	213000	209420	3580	1.02
Maryland	276000	211590	64410	1.30
Massachusetts	90000	77390	12610	1.16
Minnesota	1104800	759990	344810	1.45
Mississippi	1700000	1767950	−67950	0.96
Missouri	1600000	1071110	528890	1.49
Nebraska	200000	597830	−397830	0.33
New Hampshire	93417	92070	1347	1.01
New Jersey	161509	129420	32089	1.25
New York	940000	554740	385260	1.69
North Carolina	1111000	1285590	−174590	0.86
Oklahoma	425000	1014740	−589740	0.42
Rhode Island	13000	15990	−2990	0.81
South Carolina	800000	807740	−7740	0.99
Texas	3367200	3385920	−18720	0.99
Vermont	123000	111630	11370	1.10
Virginia	1000000	682330	317670	1.47
West Virginia	761000	561530	199470	1.36
Wisconsin	1112300	2119670	−1007370	0.52
Total	20354137	20429770	−75633	1.00

**Table 3:** Comparison between four annual estimates reported by the Southeast Deer Study Group during 2001 to 2005 and the 2003 map estimate, with estimates reported by Quality Deer Management Association for consideration (Adams & Ross, 2015).

State	2001– 2002	2002– 2003	2003– 2004	2004– 2005	Mean	SD	2003 estimate	Difference	Quotient	2005 QDMA
Alabama	1750000	1750000	1750000	1750000	1750000	0	1313020	436980	1.33	1750000
Arkansas	1000000	1000000	750000	750000	875000	144338	686810	188190	1.27	N/A
Florida		800000	800000	800000	800000	0	525210	274790	1.52	N/A
Georgia	1200000	1200000	1200000	1100000	1175000	50000	1191410	−16410	0.99	1470000
Kentucky	850000	850000	900000	900000	875000	28868	518650	356350	1.69	847911
Louisiana	1000000	1000000	1000000	1000000	1000000	0	1273790	−273790	0.79	750000
Maryland	240000	296000	264000	242000	260500	26045	211590	48910	1.23	276000
Mississippi	1500000	1500000	1625000	1625000	1562500	72169	1767950	−205450	0.88	1700000
Missouri		1000000	1000000	1000000	1000000	0	1071110	−71110	0.93	1600000
North Carolina	1100000	1000000	1080000	1111000	1072750	50169	1285590	−212840	0.83	1111000
Oklahoma	475000	475000	500000	500000	487500	14434	1014740	−527240	0.48	425000
South Carolina	1000000	1000000	900000	800000	925000	95743	807740	117260	1.15	800000
Tennessee	999000	990000	990000	881500	965125	55911	736820	228305	1.31	N/A
Texas	3776052	3826146	4007748	3915862	3881452	102148	3385920	495532	1.15	3367200
Virginia	900000	970000	1000000	950000	955000	42032	682330	272670	1.40	1000000
West Virginia	940000	965000	848000	901000	913500	50993	561530	351970	1.63	761000
Southeast					18498327		17034210	1464117	1.09	N/A

## Discussion

This research demonstrated automated methodology for digitization of thematic maps to accessible GIS layers. The methods presented here are effective at reclaiming a variety of thematic maps without associated data or GIS layers. Any application of software, whether eCognition or GIMP, will assist in reducing the amount of time needed to select, draw, and edit features to digitize a map image. In part, we used object-based image analysis to delineate homogenous objects that otherwise are digitized by hand. Although object-based image analysis is applied for classification of optical imagery, it is not yet a familiar tool for the GIScience community (Chen et al., 2018), and automation is not widely known as an option for digitization. Therefore, automated digitization is not being applied to published maps without GIS layers. A published map does not have to be historical to be lacking a GIS layer; for example, the QDMA deer density maps are from the 2000s. Many historical and relatively current publications do not have archived datasets and the necessity to digitize map images highlights the value of archiving data.

It is not possible to fully analyze information in printed maps without access to that information in the form of GIS layers. We were able to estimate and compare white-tailed deer distribution and population densities and spatial change between approximately 1982 and 2003 in the conterminous US by using digitized maps, which we will archive (https://www.fs.usda.gov/rds/archive/). The described method can reduce the time and effort needed to retrieve other valuable datasets stored in thematic maps.

The value of 17 million deer for the 1982 map is in line with 15 million deer by 1978 and 26 million deer by 1993 (Miller, Muller & Demarais, 2003). During 1982, deer densities were 3.6 deer/km^2^ where deer were present and during 2003, mean deer density was 5.4 deer/km^2^ where deer were present, given a population of about 30 million. Six states had deer densities ≥9.8 deer/km^2^ during 2003. White-tailed deer had a distribution of 5.6 million km^2^ during 2003. Deer expanded about 810,000 km^2^ between 1982 and 2003, primarily in the western US because deer already were present throughout most eastern states. Nonetheless, Seton (1909) included part of Washington, Oregon, Colorado, Texas, New Mexico, and Arizona as historical white-tailed deer range where white-tailed deer are not present, according to the 2003 map, suggesting continued expansion will occur into western states. Idaho and Wyoming now may have greater white-tailed deer range than in the past.

Even with interest in deer as game species and as ecological drivers and strong investment in monitoring deer harvest and modeling deer populations, no agency is curating current and past deer population estimates from every state, particularly spatial information. The QDMA compiles estimates into reports, and similarly other organizations informally accumulate information by state from state agencies. In contrast, the Southeastern Cooperative Wildlife Disease Study coordinates among state agencies to gather information about feral swine (*Sus scrofa*) and provides information through the National Feral Swine Mapping System (NFSMS; Corn & Jordan, 2017).

### Ecological impact of changing deer densities over time

The pre-Euro-American settlement population of white-tailed deer likely was at minimum 24 million to 33 million animals in North America, mostly concentrated in the eastern US (McCabe & McCabe, 1984). However, the deer population varied over time and Adams & Hamilton (2011) calculated a population of 9 million to 19 million animals before 1500, after which diseases reduced Native American populations. Conservative estimates of white-tailed deer densities generally ranged from 3 to 8 deer per km^2^ in North America (McCabe & McCabe, 1984); therefore, deer populations historically may have been greater than 40 million animals at 8 deer/km^2^ in the eastern United States with additional millions of deer in the western US, based on still conservative estimates (McCabe & McCabe, 1984). To place a maximum bound using liberal estimates of deer densities before Euro-American settlement, deer abundance may have reached 65 million to 80 million in North America, given moderately low densities of 10 to 12 deer per km^2^ in most of the eastern US, with moderately high to high density landscapes of 15 to 20 deer per km^2^, and low densities of 3 to 4 deer per km^2^ throughout the rest of the deer distribution.

Exploitation of white-tailed deer for commercial markets by Euro-American settlers reduced the deer population to about 12 to 14 million animals by 1800 and 300,000 to 500,000 animals by 1900 (McCabe & McCabe, 1984). Commercial hunting ended due to changed public attitudes, enforced state harvest restrictions, and banned interstate shipment of illegally caught animals by the federal Lacey Act of 1900 (McCabe & McCabe, 1984). The population may have recovered to about 6 million by 1948, 15 million by 1978, 26 million by 1993, and 30 million animals by 2000 in the US (McCabe & McCabe, 1984; Miller, Muller & Demarais, 2003; Webb, 2014). Since 2000, the deer population has been relatively stable, with potentially a slight decrease due to habitat loss and degradation, hunting pressure, severe weather events, and disease may be decreasing deer populations (Webb, 2014; Adams & Ross, 2015). A white-tailed deer population of 30 million may be at or even below historical levels in most of the US, based on a wide range of historical deer population estimates.

Since Euro-American settlement, comprehensive changes in vegetation have occurred. Generally, fire-tolerant open oak and pine forests with an herbaceous vegetation ground layer have transitioned to closed forests, comprised of diverse fire-sensitive tree species and tree layers throughout the vertical profile, typically replacing the herbaceous ground layer (Hanberry, Bragg & Hutchinson, 2018). The concurrence of increased tree recruitment and decreased deer populations during Euro-American settlement may suggest that relief from browsing pressure was influential in releasing tree growth because herbivores are potential drivers of vegetation structure. However, transition back to open forests is not occurring after resumption of deer pressure at or above thresholds of 3 to 9 deer/km^2^ expected to cause change, even where deer densities exceed 10 deer/km^2^ (e.g., in Mississippi, Hanberry et al., 2014; Hanberry, Coursey & Kush, 2018; Hanberry & Abrams, 2019). Overall, research indicates that deer reduce regeneration of tree seedlings (Habeck & Schultz, 2015; Ramirez, Jansen & Poorter, 2018), but most tree seedlings will not survive with or without herbivores due to density-dependent mortality. Indeed, if densities are within or lower than historical densities, then the level of herbivory may be tolerable to plants that co-existed with historical deer browsing.

Based on analysis using these digitized maps, deer densities do not appear to be correlated with tree stocking (i.e., percent occupied growing space accounting for both density and diameter) at landscape scales in the eastern US (Hanberry & Abrams, 2019; to account for time lag of effects, we used 1982 and 1996 deer densities and current tree stocking after about 30 years and 15 years of deer browsing). It may be that when tree regeneration is limited by fire, deer and other herbivores can help maintain open forests, grasslands, and shrublands. In addition, despite variable browse preferences, almost all tree species, including species such as northern white cedar (*Thuja occidentalis*) identified as preferred browse reduced by deer browsing, have increased in relative abundance of trees (diameter ≥12.7 cm) in the eastern US between the 1800s and the current decade of 2010 (Hanberry, Palik & He, 2013; Hanberry & Abrams, 2019; Hanberry & Dey, 2019). Increases in most tree species probably preceded increases in deer densities, but recent trends during the past decades are similar to historical trends (Hanberry, 2019; northern white cedar increased slightly in northern mixed forests, B Hanberry, pers. obs., 2019). Decreasing tree species include fire-tolerant oak and pines, some wetland species, and species that are affected by novel diseases perhaps in combination with forestry practices (Hanberry, Palik & He, 2013; Hanberry & Nowacki, 2016; Hanberry & Abrams, 2019). Although woody plants have benefitted from conditions during the past century, deer herbivory is an additional stressor on herbaceous plants that have become less abundant due to intense competition with woody species; with limited growing space, abundance of forbs and grasses necessarily decreases as tree densities increase (Hanberry, Bragg & Hutchinson, 2018).

## Conclusions

Ecologically valuable published data may not be accessible except as thematic images. The functionality of thematic maps can be increased by digitizing pictures into Geographic Information Systems (GIS), or computer-readable layers. Digitized data facilitates access to and analysis of geospatial information that is relevant but relatively inaccessible. We documented a method to digitize maps into GIS layers, while providing valuable information about change in white-tailed deer population, densities, and range for the conterminous United States. These methods can be applied to other thematic maps to increase availability and recapture information stored in images.

##  Supplemental Information

10.7717/peerj.8262/supp-1 Supplemental Information 1 Digitized layer of the Quality Deer Management Association 2001 to 2005 (i.e., 2003) map of deer density

10.7717/peerj.8262/supp-2 Supplemental Information 2 Digitized layer of the Quality Deer Management Association 1994 to 1999 (i.e., 1996) map of deer density

10.7717/peerj.8262/supp-3 Supplemental Information 3 Digitized layer of the Southeastern Cooperative Wildlife Disease Study 1982 map of deer density

## Additional Information and Declarations

### Data Availability

The following information was supplied regarding data availability: The digitized maps are available in Fig. 1. The shape file data is available in the Supplemental Files and the shapefiles are available at: Hanberry, Brice B; Hanberry, Phillip. 2019. Digitized white-tailed deer densities for the continental United States. Fort Collins, CO: Forest Service Research Data Archive. DOI 10.2737/RDS-2019-0053.
